# Indents

**Published:** 1904-04-15

**Authors:** 


### INDENTS.

☞ **Brief Articles of Technical or Scientific Value will receive notice and comment. Contributions invited.**

**Exposure of Gold Band.** Every artistic prosthetician knows that a durable banded crown may be constructed, having little or no gold in evidence. Unless this fact be observed and adhered to, the banded crown would best be known as the "abandoned" crown, for it certainly is an abomination in the eyes of the discerning.—J. T. Grant, in *Pacific Gazette.*

**Improving the Status of Our Profession.** To do this let us get closer together—like a solid phalanx—let us work more in unison; let us abolish our cut-rate business methods, put aside our petty jealousies, and "quit our meanness," as Sam Jones says. To do all this may not be possible, but among men who are above the methods and policies of cheap-john commercialism, most of this is possible. Our formula to bring about this happy result would be to get together and cultivate reciprocal relations and exchange ideas and experiences.—Editorial in *Texas Journal.*

**Aluminum Lining for Rubber Plates.** A simple method is as follows: Take chloroform, three ounces; carbon disulphid, one ounce; powdered aluminum, one ounce. Before mixing the ingredients make a saturated solution of the chloroform and white vulcanite rubber. When the case is ready to pack, give the model a good coat of collodion, and follow this with about three or four coats of the aluminum preparation. I have used this for about six years, and have one plate under observation made in this way five years ago, and can see no change in it.—Dr. B. L. Conway, *Brief.*

**Opening Pyrozone Tubes.** Place the tube up to its neck in an ice-water bath until the paper label can be easily rubbed off. Remove from the bath, dry and place into a proper sized hole in a large cork which fits the mouth of a good sized bottle. Have the large bottle dry so that if the pyrozone tube breaks it will not dilute the pyrozone. Place the cork with pyrozone tube into the bottle leaving only the neck of the pyrozone tube outside. Take a sharp file and nick the tube at the point indicated, and it can be readily handled, as the cork holds it steady. Should the pyrozone explode the liquid will be caught in the large bottle and saved.—Dr. F. H. Field, in *Items of Interest*.

**Soft Water a Cause of Carious Teeth.** A writer in the *Referee* says that in some portions of Lancashire, England, you will find towns in which edentulous mouths are the rule. An excursion party was inspected in Blackpool and an average of these teeth was found in the company made up of men, women, lads and lassies. I was told that in the district from which these people came, the drinking water was devoid of mineral or calcic elements, and the teeth simply crumbled away in childhood. In Glasgow, which is supplied with soft water from Loch Katrine, the people's teeth are notoriously bad, while in Birmingham where the water is hard, the teeth are of better structure and more durable.—*Record*.

**Fusible Metal for Use in Crown-work.** Following is the formula for a fusible metal, fusing at about 165 degrees. This metal will not stick to the gold when heating it to separate the fusible metal from the gold crown in making seamless crowns:

| | |
|---|---|
| Cadmium | 26 6-10 grammes |
| Bismuth | 94 8-10 grammes |
| Lead | 38 8-10 grammes |
| Tin | 40 grammes |

Should a film of metal adhere to the crown, chill the crown in water, when the metal can be peeled off without trouble. The crown should be finally boiled in dilute nitric acid to free it from all contamination.—Dr. C. E. Post, *Gazette.*

**The Future of Platinum.** The *Chemische Zeitung* says that in 1888 the price of platinum was 390 marks ($93.60) per kilogram, or about $3.61 per ounce. In 1900 the price had risen to 2000 marks ($840) per kilogram, while to-day it is quoted at about the price of gold. The total amount mined this year will be in the neighborhood of 6000 kilos (about 16,080 pounds troy), while the demand will be in the neighborhood of 7500 kilos (about 19,290 pounds troy). Until recetnly the excess of consumption over production was met by using old material, apparatus that had gone out of use, etc., but this has reached its limit and unless new deposits of platinum ore are discovered, or a substitute for the metal is discovered, the price will continue to advance.

**Dental Stage Properties.** The severe economy which had to be exercised by the management of the Norwegian Theater in Bergin, in 1849, is illustrated by a story told by Mr. William Archer in the current number of the *Fortnightly Review.* A lady had been engaged for the part of "second old woman" when it was discovered that her elocutionary powers were impaired by the fact that she had lost one of her front teeth. Impoverished as she was the management came to her rescue, and bore the expense of the necessary dentistry. When she retired, however, after two seasons she had to leave the tooth behind her, the example of the dentist's art being the property of the theater. The management were too poor to part with it.—*Record.*

**Pressure Anesthesia.** Syringe the cavity with a warm solution of lysol, one to one thousand, to sterilize it. Then place the dam in position, dry the cavity and place in the cavity a pledget of cotton saturated with a solution of cocain in adrenalin, one part cocain to five parts of the adrenalin solution. After five minutes remove the application and with instruments remove all debris and decayed dentine and uncover the pulp. Wipe out the cavity with absolute alcohol to sterilize and apply the cocain adrenalin mixture. As usual and by slow but steadily increasing the pressure the entire pulp will become anesthetized. Remove the pulp by proper instrumentation and cleanse with hydrogen dioxide, dry and fill permanently. I use as an antiseptic lubricant a saturated solution of thymol in oil of cloves.—Dr. DeVries in March *Items*.

**Supporting a Split Tooth.** Dr. C. E. Kells, in the *Items of Interest*, describes this method of bolting together teeth that have been split through their crowns. A bicuspid crown which had been fractured was drawn to place by a brass wire ligature, and a hole drilled through it of proper size to admit a threaded iridio-platinum wire, which had a cone-shaped nut on each end. The buccal and labial ends of the hole were counter sunk to exactly accommodate the cone-shaped nuts. The wire was then placed in position, the hole having been saturated with an antiseptic solution of gutta-percha, and the nuts screwed firmly to place. The brass ligature wire was then removed and the nuts polished, so that they appeared as small gold fillings, the buccal one only being visible. Eighteen months have passed since the operation and it seems to be in as good condition as when first made.

**Matrix, Adaptation with Putty.** When the cavity is prepared cut the matrix material to proper size and form. Place in position and press to general form of cavity with fingers or slight instrumentation, and stretch loosely over it a strip of thin dam, then take a small quantity of ordinary putty, rather stiff, and lay it on the dam and over the cavity and press into the cavity with the fingers or suitable spatula, gently at first until the matrix material has been carried into the bottom of the cavity and then put considerable force on it and carry it into every part of the cavity. Withdraw the pressure and let go of the rubber strip and its elasticity will lift the putty from the cavity and leave the matrix in position. Final adaptation if necessary may be made with burnishers as usual. This will not usually be necessary for the gold matrix.—Dr. W. Holloway, in March *Items*.

**Formaldehyde in Milk.** Prof. Tanzer in his experiments made tests to determine a small quantity of formaldehyde in milk, watered, milk two days old and milk two weeks old. In each experiment he was successful. Later these tests were verified by standard means, and it is thought that Prof. Tanzer has made a valuable scientific discovery, as he proved to the entire satisfaction of the gentlemen present that instead of formaldehyde disappearing when administered in small doses, it is retained in part, and with a continuation of the doses a sufficient amount would accumulate to cause death.

In all probability, a public test will soon be made before the commissioners. It has been suggested that newly-born kittens will be used for the tests, some having been raised on the pure milk and others on milk containing formaldehyde.—*Pacific Drug Review*.

**Bridge Expedient.** In cases where the teeth have lost their perpendicular positions and lean toward each other, precluding the adjustment of the piercaps parallel to each other, as in bridging from a tilted cuspid to a tilted molar, both teeth having living nerves adjust an opened-faced band on cuspid and a cap crown on the molar in position for occlusion and attachment of bridge. Bend or stamp a piece of stiff plate to fit for a short distance on the gum in front of the molar and to posterior crown, solder to it. Solder a cylinder of proper form and size to telescope into a shell cap crown suitable for the position parallel to the cuspid. Then add dummies sufficient to complete the span and solder together and to the cuspid pier as well as to the crown that is to act as the distal pier and which telescopes over the cylinder soldered on the plate projecting from the posterior crown.—C. B. Dalby, in *Dental Record.*

**Injecting Pulps.** Experiencing difficulty in getting a secure anchorage for the hypodermic needle point in some pulps, and consequently being unable to exert sufficient pressure to force the cocain into the pulps, I reconstructed my needle point to meet such cases. A small section of cone shaped metal tubing of the proper size to fit snugly the reinforced hypodermic needle, is soldered to the end of the needle with the floring end outward or toward the point of the needle. A disk of thick unvulcanized rubber of ample size to cover the entire exposure is made and with the dam punch a hole is made in the center. The cavity is dried and exposed region covered with cavity lining or chloro-percha and the soft rubber disk applied with the hole in its center directly over the exposure. The syringe is now filled with cocain solution and its bell shaped point place over the exposure on the soft rubber disk and pressure gradually applied.—J. A. Johnson, in March *Items.*

**Echafalta for Stomatitis.** Large ulcers formed in the mouth and throat; the patient had eaten almost nothing for a week, and could scarcely speak. Dissolved four drams of echafalta and four drams of glycerine in four ounces of distilled water, and directed the patient to keep a pledget of cotton saturated with this preparation in the mouth. The result was very beneficial at the end of thirty-six hours when the bottle of medicine was exhausted. The prescription was refilled and in two days the mouth was well.—R. L. Chase.

Dissolve three drams of echafalta in three ounces of water and inject in pyorrhea pockets, and administer internally three times a day a teaspoonful of a solution of two drams of echafalta in four ounces of water. A few drops of echafalta on the toothbrush will stimulate the circulation and nutrition, and act as an antiseptic. Should be used after the usual mouth toilet of brushing the teeth with powder.

**Mouth Alcohol Heater.** Some years since some one suggested a tiny alcohol lamp for use in the mouth in softening gutta-percha under crowns, especially in the upper teeth. This little affair was made of a glass vial, cork and bit of cotton twine, the last pinched between the cork and neck of the vial. Burning the cork was prevented by covering with a layer of gold foil, a suggestion by Dr. W. W. Belcher. This was and is an emergency appliance. Something of the kind should be on hand and ready for use at any time, for any other as well as for the purpose specified. To that end we have had resort to the miniature oilers—pocket affairs—on sale as a rule by dealers in bicycle supplies. These equipped with alcohol and a bit of loosely wound cotton thread, such as may be pulled from the candle wick of yore, make the "addition to the dental armamentarium" complete. The slight curving of the tip or burner, which should be of extra length, adds very materially to the convenience of application to the parts to be softened.—*Office and Laboratory.*

**Building Dental Societies.** We must draw on every man and make him feel that he is responsible for something in the profession. I would like to see a law passed that if a man did not join the State society he could not practice in the State, then we would get them all in and have a good society. But we cannot do that. When we impress upon the mind of every man entering the profession, that he owes something to his neighbor as well as to himself, then we will get our society built up. You will find men in every place in the United States that are doing active work for their societies. This work should not fall on one or two men, but every dentist should feel that he has something to do, if it is only sending in his dues to the society.

If our journals would take up this subject, and have in every issue something pertinent to this question, until every man who reads a dental journal would feel that he has something to do, that he has something to say, and that a responsibility was resting upon him, then we would see our societies built up.—E. C. French, in *Review.*

**Boiling Points of Metal.** By the use of vessels of quartz heated by an electric furnace, Krafft has determined the boiling point of certain metals, as follows: Zinc sublimes below 300 degrees, and at 640 degrees distills fairly quickly; the corresponding temperatures for cadmium are 322 degrees and 448 degrees. Selenium distills quickly at 380 degrees; tellurium at 550 degrees, boiling being observable at 535 degrees. Lead boils rapidly and distills at 1,160 degrees. Tin proved very refractory, no distillation occurring even at 1,100 degrees. At 605 degrees antimony sublimes slowly, and at 775 degrees to 780 degrees rapidly. Sublimation of bismuth commenced at 540 degrees, the sublimate assumed the form of drops at 930 degrees, and the metal boiled briskly at 1,050 degrees. A slight mirror of silver appeared at 1,090 degrees, and rapid vaporization proceeded at 1,340 degrees. Copper and gold boil at too high temperatures to be examined, even in silica; with the former a slight amount of sublimate formed at 1,315 degrees, and with the later extremely little vapor arose even at 1,375 degrees, which is near the point at which the resistance of silica breaks down.—*Pacific Medical Journal.*

**The Vacuum Chamber.** For the form use a metal composed of equal parts of lead and pure tin. This nesses are all that are required. Always cut a new form for each case. It should never reach nearer than a quarter is rolled in sheets of 26, 28 and 30 gauge. These three thick-of an inch the heel or back edge of the plate. It should extend forward sufficiently to prevent rocking on the hard part of the palate. It should have no square edges, but with a fine file be nicely beveled. It should be broad enough to cover the horizontal portion of the hard palate. Do not give it a heart, or any other fancy shape, but let it conform to the general form of the arch of the jaw. After the metal form is pressed down smoothly and fastened to the model, in the position desired, on either side of the hard part of the palate, scrape a little plaster from the model, extending this slight change a little farther in the rear than the plate will reach, so there will be a snug pressure in the soft tissue at those points when the finished denture is placed in the mouth.

A good many dentures made with the air chamber in the manner outlined have been worn from twenty to thirty years, some of them intended as temporary plates, and all of them free from any morbid condition and have "held up" to the satisfaction of all concerned.—*Office and Laboratory.*

**Oxpara in Putrid Canals.** The canal should first be cleansed and sterilized. And in doing this care should be exercised so that apical irritation may be avoided. Open freely to the canal and flood the putrid pulp with hydrogen-dioxide and let it remain for a few minutes or until effervescence has ceased. With a clean, sharp broach. remove the soft contents of the canal, being careful not to force any through the apex of the root with the broach Pass the broach down the canal wall and then draw it out in the mass of pulp tissue. Wash the canal again with hydrogen-dioxide and dry carefully so as to determine if any remnants may be still clinging to the walls. When thoroughly cleansed, mix the oxapara powder and liquid to a thick solution with a spatula on a glass slab and introduce into the canal with a suitable probe. If the tooth is to be filled permanently at this time, when the canal has been filled with the oxapara paste, take a gutta-percha cone on the end of a suitable canal plugger and force it into the canal as far as possible. Then insert the filling over the canal filling. Unless there has been apical infection the tooth will be comfortable and recurrent pericementitis will be avoided. It would be better to make one or two temporary dressings of the oxapara paste before permanently sealing in cases of pericemental infection of long standing.

**Lining of Rubber Dentures with Aluminum.** For the solvent use chloroform one part, carbon disulphid, one part, and naphtha one part; for powder, the aluminum used by plumbers in bronzing metal work.

Pack the case as usual, using the flask-press, and the wet cloth to test the pack. An excess of rubber is fatal to the best results. The flask should be made to come together under gentle pressure.

The palatal surface of the denture should be made of as large pieces as possible. This gives a smooth surface and prevents penetration of the mass by the lining, an event certain to mar the appearance of the palatal surface of the denture.

After packing a swab of cotton wound on a wood toothpick is dipped into the solvent and applied to the palatal surface of the pack and a small quantity of the powder dusted on to this moistened surface and rubbed in with the swab again saturated with the solvent. This procedure is continued until the surface refused to hold more. A new swab was then made and used dry, and with it dry aluminum is applied to the model.

The flask is then closed and bolted and the case vulcanized. With the vulcanization completed the flask is allowed to come "stone cold" before opening. Without this precaution the lining would adhere in part to the model.—C. W. La Selle, in *Office and Laboratory*.

**Gutta-percha Plates.** Dr. W. O. Talbot describes in the December *Northwestern Journal* a method of making plates of vulcanizable gutta-percha. Prepare plaster models and articulate them as for a rubber case. Warm a sheet of vulcanizable gutta-percha and adapt to the models as you would wax for a base plate, and cement it around the edges to the model with a rubber cement made by dissolving rubber in gasoline. Carefully remove all wax from the pins of the teeth with chloroform on cotton. Warm the cervical ends of the teeth and set them in position on the base plate, and attach the pins by warming small pieces of the gutta-percha on a spatula. Care must be exercised to prevent burning it, as this would prevent its vulcanizing. After all the teeth are set and built up on the lingual surface to contour by warming and pressing to shape with the fingers, it should be cooled in water and tried in the mouth. The plate is then replaced on the model and again cemented to place. The teeth are then, one by one, taken off leaving their imprint in the gutta-percha so that they may be subsequently replaced. A strip of pink rubber is then cut long enough to cover the extent of the labial face of the plate, and wide enough to extend from the imprint of the pins in the gutta-percha to the edge of the labial rim. This is warmed and pressed to place, and the teeth are then replaced. Each tooth is warmed as before at its cervical end and pressed down into the warm pink rubber, displacing it until it fits into its own imprint. If carefully done, a nice festoon of the pink rubber will be thrown up around the cervical and labial ends of the teeth, giving a life-like appearance and requiring little finishing. With a warm spatula the labial surface is made smooth and the gutta-percha forced in around the pins and finishing ridges of the teeth. If desired, the whole lingual surface may be covered with pink rubber. The case is then invested by imbedding it in plaster in the flask. As there is no wax to remove, the flask is not separated, but bolted together at once and vulcanized as soon as the plaster has set. The plates should be vulcanized longer than rubber. Good results will be secured by vulcanizing one hour and forty minutes at 310 degrees. It is claimed that gutta-percha plates are denser than rubber and will be tolerated more kindly by the mucous membrane. It remains to be seen if they are as durable.

